# Validation of the 9th Edition of the TNM Classification in Patients with NSCLC and Lymph Node Involvement: A Retrospective, Multicentric, Observational Study

**DOI:** 10.3390/cancers18040702

**Published:** 2026-02-20

**Authors:** Carolina Sassorossi, Marco Chiappetta, Filippo Lococo, Gloria Santoro, Pierluigi Novellis, Giulia Veronesi, Riccardo Di Fonzo, Filippo Tommaso Gallina, Francesco Facciolo, Vittorio Aprile, Alessandra Lenzini, Marco Lucchi, Sara Ricciardi, Giuseppe Cardillo, Andrea Tornese, Ludovic Fournel, Marco Alifano, Stefano Margaritora

**Affiliations:** 1 Unit of Thoracic Surgery, Fondazione Policlinico Universitario A. Gemelli IRCCS, Università Cattolica del Sacro Cuore, 00168 Rome, Italy; filippo.lococo@policlinicogemelli.it (F.L.); stefano.margaritora@policlinicogemelli.it (S.M.); 2 Thoracic Surgery, AOU Renato Dulbecco, Magna Graecia University, 88100 Catanzaro, Italy; marcokiaps@hotmail.it; 3 Department of Surgery, Fondazione Policlinico Universitario A. Gemelli IRCCS, 00168 Rome, Italy; gloria.santoro@policlinicogemelli.it; 4 Department of Thoracic Surgery, IRCCS Ospedale San Raffaele, 20132 Milan, Italy; pierluigi.novellis84@gmail.com (P.N.); veronesi.giulia@hsr.it (G.V.); difonzo.riccardo@hsr.it (R.D.F.); 5 Thoracic Surgery Unit, IRCCS Regina Elena National Cancer Institute, 00144 Rome, Italy; filippogallina92@gmail.com (F.T.G.); francesco.facciolo@gmail.com (F.F.); 6 Thoracic Surgery, Department of Surgical, Medical and Molecular Pathology and Critical Care, University of Pisa, 56126 Pisa, Italy; aprilevittorio@gmail.com (V.A.); alessandralenzini2@gmail.com (A.L.); m.lucchi@med.unipi.it (M.L.); 7 Unit of Thoracic Surgery, Azienda Ospedaliera San Camillo Forlanini, 00152 Rome, Italy; ricciardi.sara87@gmail.com (S.R.); gcardillo@scamilloforlanini.rm.it (G.C.); 8 Unit of Anatomy and Pathological Histology, Azienda Ospedaliera San Camillo-Forlanini, Carlo Forlanini Hospital, 00151 Rome, Italy; atornese@scamilloforlanini.rm.it; 9 Department of Thoracic Surgery, Cochin Hospital, Paris-Descartes-University, 75014 Paris, France; ludovic.fournel@aphp.fr (L.F.); marco.alifano@aphp.fr (M.A.)

**Keywords:** NSCLC, IX edition TNM, nodal involvement

## Abstract

Background: The ninth edition of the TNM classification introduced refinements in nodal staging, subdividing mediastinal N2 disease into N2a (single-station) and N2b (multi-station) involvement, alongside several stage group adjustments. The aim of this study is to validate the new TNM in patients with nodal involvement who underwent surgery. Methods: This is a multicentric, retrospective study including NSCLC patients with pathological N1 or N2 involvement who underwent anatomical pulmonary resection between January 2020 and December 2023. Clinical, surgical, and pathological data were collected, including tumor characteristics, lymphadenectomy details, and adjuvant therapy. Patients were reclassified according to the ninth TNM groups: stage IIB, including T1N1, T2N1, and T3N0; IIIA, including T1N2b, T2-3N2a, T3N1, T4N0, and T4N1; and IIIB, including T2-3N2b, T4N2a, and T4N2b. Overall survival (OS) and disease-free survival (DFS) were analyzed using Kaplan–Meier curves. Results: The final analysis involved 291 patients. The three- and five-year OS rates were 82% and 71% for stage IIB, and 75% and 58% for stage IIIA, respectively. At stage IIB, a significant difference was found only for DFS comparing T2N1 and T1N2a, whereas stage IIIA showed no significant differences in either OS or DFS, confirming its prognostic homogeneity. Conversely, stage IIIB exhibited significant heterogeneity in survival (OS *p* = 0.031; DFS *p* < 0.0001), with T4N2b subgroups showing the worst outcomes compared to T2-3N2b and T4N2a. Conclusions: Our validation of the ninth edition of lung cancer staging shows improved prognostic granularity: IIB shows non-homogeneous DFS, IIIA is homogeneous, and IIIB shows major OS/DFS substage differences, warranting analyses with larger samples for refined stratification.

## Simple Summary

The ninth edition of the TNM classification introduced refinements in nodal staging for non-small-cell lung cancer (NSCLC), particularly the subdivision of mediastinal N2 disease into single-station (N2a) and multi-station (N2b) involvement, with corresponding changes in stage grouping. This multicenter retrospective study aimed to validate the prognostic performance of the new TNM system in surgically treated patients with nodal involvement. A total of 291 non-small-cell lung cancer (NSCLC) patients with pathologically proven N1 or N2 disease who underwent anatomical pulmonary resection between 2020 and 2023 were analyzed. Patients were reclassified according to the ninth TNM edition, and overall survival (OS) and disease-free survival (DFS) were evaluated. Stages IIB and IIIA showed largely homogeneous prognostic behavior, with limited differences among subgroups. In contrast, stage IIIB demonstrated significant survival heterogeneity, with the poorest outcomes observed in T4N2b disease. These findings support the improved prognostic value of the ninth TNM edition and suggest further refinement for stage IIIB.

## 1. Introduction

Lung cancer is the second most common cancer worldwide after breast cancer in women, and it remains the leading cause of cancer-related mortality [1]. The TNM staging system represents the cornerstone for therapeutic decision-making in non-small-cell lung cancer (NSCLC) [2,3].

The ninth edition of the TNM classification for lung cancer, compared with the eighth edition, did not introduce modifications for the T descriptor.

The N category definitions have remained the same since the fourth edition in 1987, and their ability to discriminate prognosis was confirmed again using the ninth edition data. Nevertheless, it has long been argued that lung cancer nodal staging does not adequately reflect disease burden, which is prognostically relevant in many other solid tumors. The use of lymph node stations, rather than lymph nodes, as the base unit for quantification has been considered solid and feasible because the enumeration of lymph nodes is less reliable. Lymph nodes with metastasis cannot be precisely counted on radiologic studies, rendering it infeasible for use in clinical staging. The variability introduced by the extent of the nodal dissection, fragmentation of nodes, and specimen handling calls into question the accuracy of any pathologic lymph node counts [4]. For instance, the current system does not differentiate between an incidental pathologic metastasis in a single paratracheal node and bulky metastatic involvement across multiple ipsilateral mediastinal nodal stations, even though both are assigned to the same N category [5].

Studies conducted during the development of the seventh and eighth editions had already suggested that disease burden within hilar and mediastinal lymph nodes was significant for the definition of prognosis [6,7]. However, the datasets available for these editions were insufficient to enable a true stratification of N based on the number of involved lymph node stations. A proposal for the eighth edition had aimed to assess the descriptors pN0, pN1, pN2a1 (skip metastases), pN2a2, and pN2b in a more robust dataset [8,9]. In the analysis supporting the eighth edition, survival curves for the skip metastasis group (pN2a1) overlapped with those for patients with involved hilar lymph nodes, failing to yield clearly distinct prognostic categories for either pathological or clinical staging [9,10].

In contrast, the proposal for the ninth edition introduced a subdivision of mediastinal nodal involvement into two categories—N2a and N2b—according to single- versus multiple-station involvement.

Additionally, several stage groupings were revised. Part of stage IIB (T1N1M0) in the eighth edition was reclassified as stage IIA (T1N1M0) in the ninth edition. Stage IIIA (T1N2AM0) in the eighth edition was divided into stages IIB (T1N2aM0) and IIIA (T1N2bM0) in the ninth edition [10].

To support implementation of the ninth edition, validation studies in single-center or multicenter settings are required, and one such study has already been published by Wang and colleagues [10]. Their analysis showed that the TNM staging in the ninth and eighth editions has similar predictive power and accuracy for the overall survival of patients with NSCLC.

However, few studies have reported the homogeneity of the new proposed subgroups. We decided to analyze our cohort using the ninth edition of the TNM staging system, assessing OS and DFS for each stage, and evaluating homogeneity among subgroups concerning survival outcomes.

## 2. Materials and Methods

### 2.1. Ethics Statement

The study was conducted in accordance with the Declaration of Helsinki of 1964 and its subsequent amendments. The study was approved by the Ethics Committee of the Fondazione Policlinico Universitario Agostino Gemelli IRCCS as the sponsor center and transmitted to the other centers (ID number 3100, 7 May 2020).

### 2.2. Patients

The study included patients with a diagnosis of NSCLC with pathologically proven N1 or N2 involvement after surgery, between 1 January 2020 and 31 December 2023, with disease staging classified according to the proposed ninth edition of TNM. Data were collected from the following centers:

- Agostino Gemelli University Polyclinic Foundation “A. Gemelli” IRCCS;
- National Cancer Institute “Regina Elena”;
- San Camillo Forlanini Hospital;
- University of Turin;
- University of Pisa;
- San Raffaele Hospital;
- Humanitas Research Hospital;
- Hôpital COCHIN, Paris.

Inclusion and exclusion criteria were as follows:

Inclusion criteria:

- Informed consent;
- Age > 18 years;
- Non-small-cell lung cancer;
- Anatomical pulmonary resection (segmentectomy, lobectomy, bilobectomy, or pneumonectomy);
- Preoperative PET CT (positron emission tomography) and CT (computed tomography);
- Pre- and postoperative discussion at multidisciplinary meeting.

Exclusion Criteria:

- Age < 18 years;
- Pregnancy;
- Psychiatric disorders;
- Atypical resections;
- Distant metastases.

Considering that anatomical resection remains the current standardized treatment and that the purpose of this work is the validation of the staging, particularly for the N parameter, only patients with complete data on postoperative staging and treated with anatomical resection were included in this analysis.

As patients were included for suspected N involvement, the correct therapeutic intervention is still anatomical resection, and only these patients were considered.

The groups of interest of the ninth edition, which included the new N classification in N2a and N2b, were classified as follows:

- Stage IIA: T1N1;
- Stage IIB: T1N2a, T2N1, T3N0;
- Stage IIIA: T1N2b, T2-3N2a, T3N1, T4N0, T4N1;
- Stage IIIB: T2-3N2b, T4N2a, T4N2b.

The following clinical, surgical, and pathological characteristics of the patients were collected: performance status, clinical stage, preoperative histology, preoperative staging, type of surgery, pathological staging, histology, presence of STAS (spread through air spaces), number of lymph nodes removed per dissected station (hilar and mediastinal), number of metastatic lymph nodes per dissected station, tumor grading, type of lymphadenectomy, and complications according to Clavien–Dindo classification. Postoperative treatments were administered according to the most recent guidelines, according to stage and histology.

The primary endpoint was to validate the prognostic role of the new proposed classification in terms of overall survival and disease-free survival. Overall survival was calculated from surgery to death, and disease-free survival was considered from surgery to radiologically detected recurrence.

The analysis was performed to evaluate the prognostic validation on the basis of survival discrimination.

### 2.3. Statistical Methods

Exploratory data analysis (EDA) was performed to characterize the cohort. Continuous variables were summarized using means, standard deviations, medians, and interquartile ranges; categorical variables were reported as absolute numbers and percentages. Survival differences among stage subgroups were evaluated using Kaplan–Meier analysis. Cox proportional hazards models were fitted to assess the influence of relevant covariates on OS and DFS. Statistical significance was set at *p* ≤ 0.05. Analyses were performed using R 4.4 statistical software.

## 3. Results

A total of 403 patients were included (Table 1), with 42% females and 58% males, and a mean age of 66.6 ± 11.6 years. Most patients had ECOG (Eastern Cooperative Oncology Group) performance status 0 (82.8%), and 41.7% were current smokers. Comorbidities included COPD (chronic obstructive pulmonary disease) (29.5%) and heart disease (36%). PET uptake was observed in the primary tumor in 93% of patients, N1 nodes in 29.3%, and N2 nodes in 15.9%. Clinical stage distribution according to the eighth edition of TNM was 41.5% stage I, 29.5% stage II, and 29% stage III. Surgical treatment was predominantly lobectomy (91%). Pneumonectomy was performed in 36 cases, and they were all c-staged IIIA, with suspected hilar nodal involvement at the CT and PET CT scan. Adenocarcinoma was the most frequent histology (67.5%), followed by squamous cell carcinoma (23.6%). Pathological analysis showed pT1–4 tumors in 49.6%, 28.5%, 13.1%, and 8.8% of patients, respectively; STAS was present in 21.1%; and lymphatic and vascular invasion were observed in 42.2% and 43.9%, respectively. Pathological stage considered according to the ninth edition of TNM was stage I for 38 patients (9.4%), IIA in 67 cases (16.6%), IIB in 89 (22.1%), IIIA in 162 (40.2%), IIIB in 40 (9.9%), and IVA in 7 (1.8%). Recurrence occurred in 42.1% of patients. At last follow-up, 52.1% were alive with no evidence of disease, 24.8% alive with disease, 4% dead of other causes, and 19.1% dead of disease. The final analysis was led on 291 patients staged IIB, IIIA, and IIIB according to the IX edition of TNM, as described in the CONSORT diagram (Supplementary Figure S1) (Table 2).

### 3.1. Disease-Free Survival (DFS) and Overall Survival (OS) Analysis

For the analysis of validation, a total of 291 patients were analyzed, including 89 with stage IIB, 162 with stage IIIA, and 40 with stage IIIB disease.

The median overall survival (OS) was 19 months (IQR: 10–35), while the median disease-free survival (DFS) was 15 months (IQR: 6–29).

**Figure 1:**
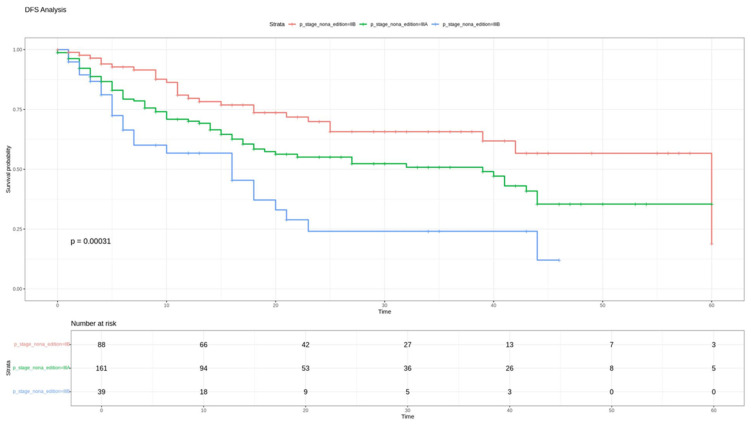
DFS for stages IIB, IIIA, and IIIB according to the IX edition of TNM.

**Figure 2:**
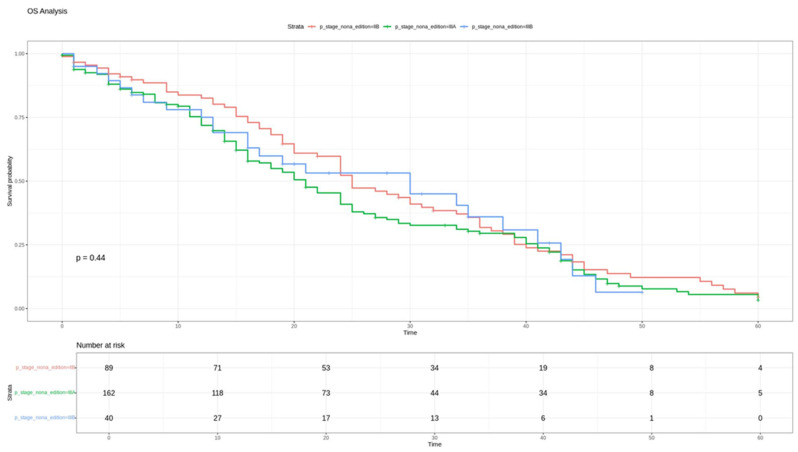
OS for stages IIB, IIIA, and IIIB according to the IX edition of TNM.

Stratification according to the ninth edition staging system demonstrated a statistically significant correlation with DFS (*p* < 0.05) (Figure 1), confirming the system’s efficacy in predicting the risk of recurrence. Conversely, the overall survival (OS) analysis using the Log-Rank test did not reach statistical significance (*p* = 0.44) (Figure 2).

Pairwise comparisons for DFS demonstrated a significantly lower risk of recurrence in stage IIB compared with stage IIIA (hazard ratio [HR] 0.62, 95% CI: 0.40–0.97; *p* = 0.035) and stage IIIB (HR 0.30, 95% CI: 0.17–0.53; *p* < 0.001). No statistically significant differences in OS were observed for stages IIIA and IIIB.

Kaplan–Meier survival analyses comparing N2a versus N2b categories according to the 9th edition staging system, regardless of T staging, were performed. The corresponding Kaplan–Meier curves did not show any difference in both OS and DFS, taking into account only the N2 parameter, without the T (see Supplementary Figures S2 and S3).

### 3.2. Prognostic Heterogeneity and Substage Survival in Stages IIB–IIIB Disease According to the IX TNM Edition

#### 3.2.1. Stage IIB

In stage IIB patients, a significant difference in disease-free survival (DFS) was observed between substages.

Patients with T2N1 disease showed a significantly higher 3-year disease-free survival (DFS) compared with those with T1N2a (78.4% vs. 42.8%, *p* = 0.0032), while no significant difference in 3-year overall survival (OS) was observed between the two groups (Table 3) (*p* = 0.044). This lack of difference underlines homogeneity among subgroups concerning OS.

**Figure 3:**
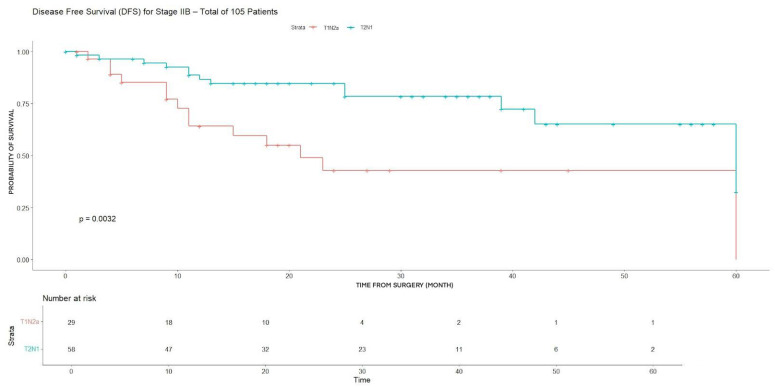
DFS for T2N1 and T2N2a (stage IIB).

**Figure 4:**
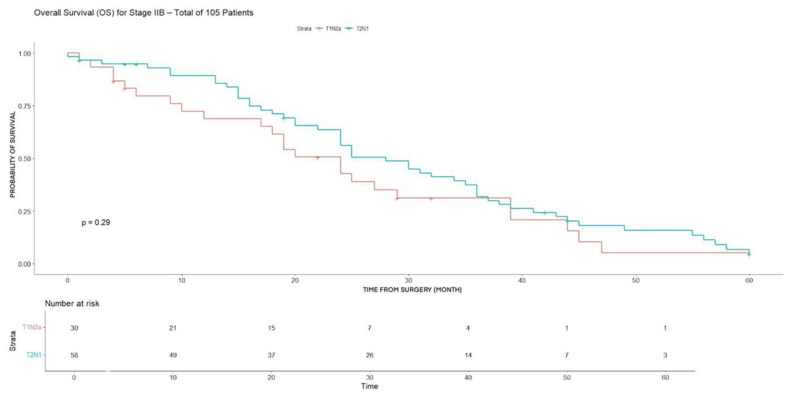
OS for T2N1 and T2N2a (stage IIB).

The same result was found when comparing DFS for T2N1 compared with those with T1N2a (*p* = 0.0032) (Figure 3). In contrast, no statistically significant difference in overall survival (OS) was detected between the two groups (*p* = 0.293) (Figure 4).

#### 3.2.2. Stage IIIA

Among stage IIIA subgroups, no statistically significant differences were found in either 3-year DFS or OS, despite some variability in DFS rates, with the highest value observed in T3N1 patients (65.9%) (Table 3).

**Figure 5:**
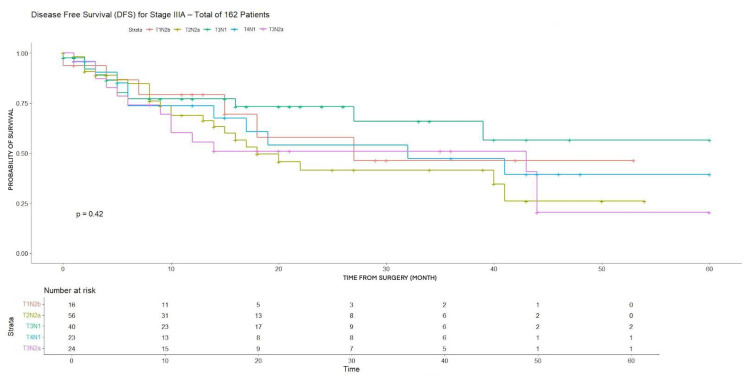
DFS for T1N2b, T2N2a, T3N1, T3N2a, T4N1 (stage IIIA).

**Figure 6:**
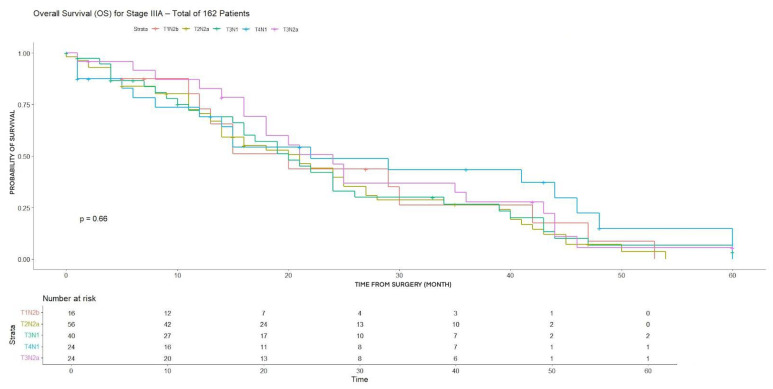
OS for T1N2b, T2N2a, T3N1, T3N2a, T4N1 (stage IIIA).

Furthermore, no statistically significant differences were observed in either DFS or OS (Figure 5 and Figure 6).

However, due to the small number of patients in some subgroups (i.e., T1N2b, 16), results have to be considered cautiously.

#### 3.2.3. Stage IIIB

In stage IIIB patients, significant heterogeneity in survival outcomes was observed across substages.

Significant differences in both 3YDFS and 3YOS were observed, with markedly worse DFS in T4N2b patients (4.7%) and significant heterogeneity in OS outcomes (*p* < 0.001) (Table 3).

**Figure 7:**
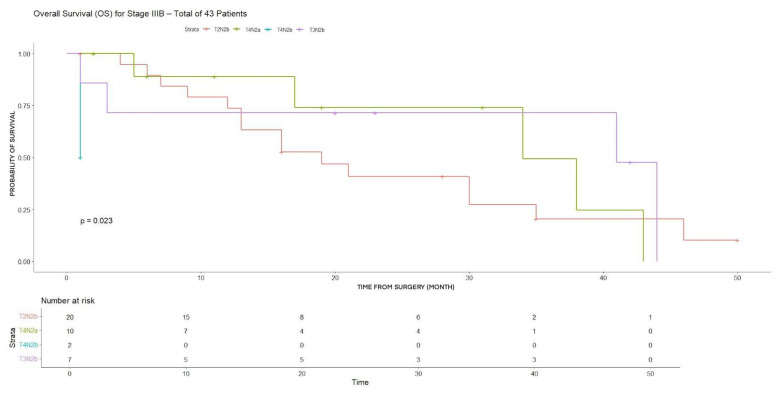
OS for T2N2b, T4N2a, T4N2b, T3N2b (stage IIIB).

**Figure 8:**
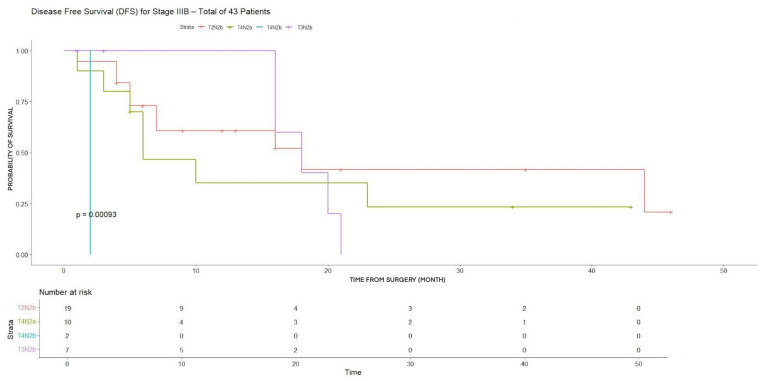
DFS for T2N2b, T4N2a, T4N2b, T3N2b (stage IIIB).

OS showed a marked and statistically significant decline with more advanced tumor and nodal status (*p* = 0.023) (Figure 7). Similarly, DFS differed significantly among T2–3N2b, T4N2a, and T4N2b groups (*p* = 0.0009) (Figure 8).

## 4. Discussion

Among the various candidate factors proposed to refine the N descriptor and improve prognostic discrimination, the IASLC (International Association for the Study of Lung Cancer) adopted the subdivision of N2 disease according to the number of involved nodal stations for the ninth TNM edition [11,12].

This distinction generated two N2 subcategories—N2a and N2b—associated with different survival outcomes and consequently incorporated into different stage groupings. Based on the prognostic divergence between single-station and multi-station N2 disease, the ninth edition introduced three downstagings and one upstaging within stages II and III [7].

The N category descriptor for lung cancer staging has remained the same since the introduction of the fourth edition of TNM in 1992 [4]; thus, there have been no changes in the distinctions among N descriptors [13,14].

In the eighth edition, a revision of lymph node (LN) metastasis assessment was proposed, distinguishing between involvement of single or multiple LN stations and the presence of skip metastases [3]. However, these more detailed N subcategories presented a major limitation, as their application was feasible only in pathological staging due to constraints in the availability and reliability of clinical information [3,4].

Based on a more comprehensive dataset that uniformly applied the IASLC lymph node map, the IASLC adopted a simplified classification in the ninth edition, differentiating only between single- and multiple-station metastases and eliminating a distinct skip metastasis category. This approach enabled improved stratification of N2 disease not only in pathological staging but also in clinical staging [15].

In line with the revised N classification, the IASLC reclassified T1N2a from stage IIIA in the eighth edition to stage IIB in the ninth edition [1]. This represents the most substantial modification in the current edition, considering that N2 disease had consistently been assigned to stage III since the second edition of the TNM classification.

The most appropriate treatment strategy remains a matter of debate due to the clinical heterogeneity of the disease and the absence of a standardized definition of resectability. In this context, surgical resection as a component of multimodal therapy for patients with resectable N2 NSCLC has gained growing relevance, particularly in the era of targeted treatments and immunotherapy [16,17,18,19,20,21].

In contrast to the reclassification of T1N2a to stage IIB, the other modifications involving upstaging or downstaging of T and N subgroups in the ninth edition were relatively modest. Although these adjustments enhanced the discriminatory performance of the ninth edition, they occurred primarily within substages (A or B) and did not result in transitions between stage II and stage III [18,19]. Likewise, the downstaging of T1N1 from stage IIB to stage IIA within stage II had no meaningful impact on diagnostic or therapeutic decision-making [20].

In our study, which included patients with NSCLC and histologically confirmed nodal involvement, we evaluated the prognostic validity of this new staging proposal. The analysis was performed to evaluate the prognostic validation on the basis of survival discrimination.

Generally, upstaging (stage migration upward) is associated with worse survival, while downstaging correlates with improved prognosis. In our cohort, we confirmed that the downstaging of T1N2aM0 from stage IIIA to IIB and of T3N2aM0 from stage IIIB to IIIA was appropriate for both OS and DFS. Conversely, the upstaging of T2N2bM0 from stage IIIA to IIIB appeared less consistent: OS and DFS in this subgroup were significantly better than in the other components of stage IIIB. In particular, patients with T4N2aM0 and T4N2bM0 exhibited the worst outcomes. These findings parallel those reported by Nakao et al. [12], who observed poor prognosis within stage IIIB for T3N2b and T4N2 disease.

Caution is needed in interpreting our results owing to the limited number of patients classified as stage III, raising the possibility that some differences may be attributable to chance. In particular, we have to underline that some subgroups were composed of few patients (i.e., T4N2b = 2)

With respect to stage IIIA, the combined effects of two downstagings (T1N2aM0 and T3N2aM0) and one upstaging (T2N2bM0) resulted in a prognostically coherent grouping, with comparable OS and DFS among subcategories. Similarly, in stage IIB, the two downstagings (T1N1M0 and T1N2aM0) resulted in a homogeneous cohort, with no differences in survival outcomes.

These findings about stage IIB and IIIA are consistent with those of Detterbeck and colleagues [21], who compared the eighth and ninth editions and highlighted the improved internal homogeneity of stage groupings, particularly for pathological staging.

As mentioned earlier, stage classification has implications not only for prognosis but also for treatment decision-making. Standardizing treatment strategies remains an unmet clinical need. According to the NCCN guidelines, the optimal management of N2 disease consists of definitive concurrent chemoradiation followed by durvalumab, or systemic therapy—often including neoadjuvant immunochemotherapy—followed by reassessment [12]. Surgery may be considered only for selected patients without progression after systemic therapy (with or without radiotherapy). Although still debated, some consensus exists supporting upfront surgery followed by adjuvant chemotherapy in carefully selected cases of single-station cN2 disease [21,22]. The long-recognized difference in prognosis between single-station and multi-station N2 underscores the importance of the ninth edition’s refinement of the N classification, which aims to harmonize treatment approaches across an otherwise heterogeneous group of patients. In recent years, several studies have reported favorable outcomes using perioperative immune checkpoint inhibitors combined with chemotherapy, particularly in patients with N2 disease [23,24,25].

Taking into account the new N2 subdivision, a work from Kim and coworkers [26] confirmed a clear prognostic separation between all categories (N0, N1, N2a, and N2b) in terms of both overall survival and recurrence-free survival. Furthermore, they found that N2a status was associated with worse survival than N1 status, which allowed for a much clearer prognostic separation between all categories compared with that of the eighth edition N classification. This is a very important achievement if compared to the previously proposed N2a1 definition (skip metastases) for the 8th edition of TNM, which was abandoned, as no differences were found with N1 status [27,28,29].

To the best of our knowledge, our cohort is the only one to demonstrate differences in both OS and DFS within the newly defined stage IIB and IIIB. Indeed, Nakao et al. [12] reported that, within stage IIIB of the ninth TNM edition, differences were observed only in overall survival (OS), while disease-free survival (DFS) did not significantly differ among the subgroups. The consistently worse prognosis of T4N2 disease in our analysis suggests that the current grouping may benefit from further refinement, possibly introducing an additional subdivision pending validation in larger datasets.

While stage IIIA displayed relatively homogeneous survival outcomes following the proposed up- and downstagings, stage IIB and IIIB encompassed subgroups with markedly divergent prognoses. Specifically, patients with T2N2bM0 demonstrated significantly better OS and DFS compared with those classified as T4N2aM0 or T4N2bM0, suggesting that the current stage grouping does not fully capture the prognostic impact of tumor burden and nodal involvement. This divergence raises concerns about the validity of pooling these subcategories under a single stage and may lead to suboptimal stratification, with implications for treatment planning.

The observed heterogeneity underscores the need for further refinement of stage IIB and IIIB, potentially through additional subdivision based on the combined effects of T and N descriptors. Such a revision could improve prognostic accuracy and better guide clinical decision-making, particularly in the era of multimodal and perioperative systemic therapies. Until larger, multicenter datasets validate these patterns, clinicians should exercise caution when interpreting stage IIIB outcomes and consider individualized assessment of T and N subcategories when planning therapeutic strategies.

### Study Limitations

The main limitation of our study is the incompleteness of clinical staging data, which prevented a robust analysis of cStage–pStage correlations. Nevertheless, because the dataset derives from a multicentric cohort, the results provide encouraging support for the validity of the ninth edition, particularly regarding the reorganization of the N2 descriptor, while also underscoring the need for additional investigation of the stage IIIB category in larger populations.

Considering that anatomical resection remains the current standardized treatment and that the purpose of this work is the validation of the staging, particularly for the N parameter, patients treated with wedge resections were excluded from the study.

We have to underline that the observation of the cumulative mortality curves revealed a paradoxical inversion between stage IIB, IIIA, and IIIB. A detailed case-by-case analysis (Boxplot of OS distribution, Supplementary Figure S4) identified four patients within the stage IIIB subgroup categorized as “long-term survivors”. These subjects were characterized by a survival time exceeding 30 months and were censored (alive) at the end of the follow-up period. Given the small sample size of the stage IIIB subgroup (N = 40), the persistence of these few subjects disproportionately shifted the group’s median survival, masking the expected biological aggressiveness of advanced-stage disease.

Considering a multivariable analysis with the evaluation of prognostic value of patient features, this has not been reported in the present study, and it is the topic of an ongoing analysis on the same cohort.

## 5. Conclusions

In conclusion, our validation of the ninth edition for lung cancer has demonstrated that the new edition allows for more granularity in prognostic stratification. Stage IIB showed discrimination concerning DFS. Stage IIIA seems to be homogeneous both for OS and DFS. Stage IIIB showed the greatest difference among substages both for OS and DFS. Further analysis of stage IIB and IIIB, with a larger sample size, is desirable in order to further define the stratification.

## Figures and Tables

**Table 1:** Overall clinic-pathological features.

		N. of Patients (403)
Sex	Female	172 (42%)
	Male	231 (58%)
Age at surgery ± SD		66.6 ± 11.6
ECOG	0	334 (82.8%)
	1	62 (15.4%)
	2	7 (1.8%)
Smoker	Yes	168 (41.7%)
	No	117 (29%)
	Former (at least 1 year)	118 (29.3%)
COPD	Yes	119 (29.5%)
	No	284 (70.5%)
Heart disease	Yes	145 (36%)
	No	258 (64%)
PET uptake T	Yes	375 (93%)
	No	28 (7%)
PET uptake N1	Yes	118 (29.3%)
	No	285 (70.7%)
PET uptake N2	Yes	64 (15.9%)
	No	339 (84.1%)
cStage VIII edition	I	167 (41.5%)
	II	119 (29.5%)
	III	117 (29)
Surgery	Lobectomy	367 (91%)
	Pneumonectomy	36 (9%)
Histology	Adenocarcinoma	272 (67.5%)
	Squamous cell carcinoma	95 (23.6%)
	Carcinoid	10 (2.5%)
	Other	26 (6.5%)
Grading	1	2 (0.5%)
	2	162 (40.2%)
	3	206 (51.1%)
	4	1 (0.3%)
	Not available	32 (7.9%)
pT	1	200 (49.6%)
	2	115 (28.5%)
	3	53 (13.1%)
	4	35 (8.8%)
STAS	Yes	85 (21.1%)
	No	318 (78.9%)
Lymphatic invasion	Yes	170 (42.2%)
	No	233 (57.8%)
Vascular invasion	Yes	177 (43.9%)
	No	226 (56.1%)
PT infiltration	Yes	138 (34.2%)
	No	265 (65.8%)
pStage IX edition	I	38 (9.4%)
	IIA	67 (16.6%)
	IIB	89 (22.1%)
	IIIA	162 (40.2%)
	IIIB	40 (9.9%)
	IVA	7 (1.8%)
Recurrence	Yes	152 (42.1%)
	No	251 (57.9%)
Status	NED	210 (52.1%)
	AWD	100 (24.8%)
	DOC	16 (4%)
	DOD	77 (19.1%)

**Table 2:** Analytical cohort clinic-pathological features.

Variable	N (%)
Sex	
Female	124 (42.6)
Male	167 (57.4)
Age at surgery, mean ± SD (years)	66.6 ± 11.6
ECOG performance status	
0	241 (82.8)
1	45 (15.5)
2	5 (1.7)
Smoking status	
Current smoker	121 (41.6)
Never smoker	84 (28.9)
Former smoker (≥1 year)	86 (29.5)
COPD	
Yes	86 (29.6)
No	205 (70.4)
Heart disease	
Yes	105 (36.1)
No	186 (63.9)
PET uptake (T)	
Yes	271 (93.1)
No	20 (6.9)
PET uptake (N1)	
Yes	85 (29.2)
No	206 (70.8)
PET uptake (N2)	
Yes	46 (15.8)
No	245 (84.2)
Surgical procedure	
Lobectomy	265 (91.1)
Pneumonectomy	26 (8.9)
Histology	
Adenocarcinoma	196 (67.4)
Squamous cell carcinoma	69 (23.7)
Carcinoid	7 (2.4)
Other	19 (6.5)
Tumor grading	
G1	1 (0.3)
G2	116 (39.9)
G3	149 (51.2)
G4	1 (0.3)
Not available	24 (8.3)
STAS	
Yes	61 (21.0)
No	230 (79.0)
Lymphatic invasion	
Yes	123 (42.3)
No	168 (57.7)
Vascular invasion	
Yes	128 (44.0)
No	163 (56.0)
Pleural infiltration	
Yes	100 (34.4)
No	191 (65.6)
Pathological stage (IX edition)	
IIB	89 (22.1%)
IIIA	162 (40.2%)
IIIB	40 (9.9%)
Recurrence	
Yes	122 (41.9)
No	169 (58.1)
Status at last follow-up	
NED	152 (52.2)
AWD	72 (24.7)
DOC	12 (4.1)
DOD	55 (18.9)

**Table 3:** Three-year DFS and OS according to pathological subgroups within each stage.

STAGE	NUMBER	3Y-DFS	*p*-VALUE	3Y-OS	*p*-VALUE
IIB-groups			0.003		0.293
T2N1	58 (66%)	78.40%		31.80%	
T1N2a	30 (34%)	42.80%		31.20%	
IIIA-groups			0.366		0.59
T1N2b	16 (10%)	46.30%		26.20%	
T2-3N2a	80 (49.4%)	41.50%		26.50%	
T3N1	40 (26.7%)	65.90%		26.70%	
T4N1	24 (13.9%)	47.30%		43.40%	
IIIB-groups			0.001		<0.001
T2-3N2b	27 (67.5%)	41.70%		20.50%	
T4N2a	10 (25%)	23.30%		49.40%	
T4N2b	2 (7.5%)	4.70%		50%	

## Data Availability

Data is available upon request.

## Supplementary Materials

The following supporting information can be downloaded at https://www.mdpi.com/article/10.3390/cancers18040702/s1, Figure S1: CONSORT diagram for patient selection; Figure S2: K-M curve for OS N2a vs. N2b; Figure S3: K-M curve for DFS N2a vs N2b; Figure S4: boxplot per long survival dispersion.

## Abbreviations

NSCLC	non-small-cell lung cancer
OS	overall survival
DFS	disease-free survival
PET CT	positron emission tomography
CT	computed tomography
STAS	spread through air spaces
EDA	exploratory data analysis
ECOG	Eastern Cooperative Oncology Group
COPD	chronic obstructive pulmonary disease
IASLC	International Association for the Study of Lung Cancer

## References

[1] Ginsberg R.J., Rubinstein L.V. (1995). Randomized trial of lobectomy versus limited resection for T1 N0 non-small cell lung cancer. Ann. Thorac. Surg..

[2] Goldstraw P., Ball D., Jett J.R., Le Chevalier T., Lim E., Nicholson A.G., Shepherd F.A. (2011). Non-small-cell lung cancer. Lancet.

[3] Asamura H., Chansky K., Crowley J., Goldstraw P., Rusch V.W., Vansteenkiste J.F., Watanabe H., Wu Y.-L., Zielinski M., Ball D. (2015). The International Association for the Study of Lung Cancer Lung Cancer Staging Project: Proposals for the Revision of the N Descriptors in the Forthcoming 8th Edition of the TNM Classification for Lung Cancer. J. Thorac. Oncol..

[4] Goldstraw P., Goldstraw P. (2009). The History of TNM Staging in Lung Cancer. Staging Manual in Thoracic Oncology.

[5] Ramirez R.A., Wang C.G., Miller L.E., Adair C.A., Berry A., Yu X., O’BRien T.F., Osarogiagbon R.U. (2012). Incomplete Intrapulmonary Lymph Node Retrieval After Routine Pathologic Examination of Resected Lung Cancer. J. Clin. Oncol..

[6] Rusch V.W., Crowley J., Giroux D.J., Goldstraw P., Im J.-G., Tsuboi M., Tsuchiya R., Vansteenkiste J., International Staging Committee, Cancer Research and Biostatistics (2007). The IASLC Lung Cancer Staging Project: Proposals for the Revision of the N Descriptors in the Forthcoming Seventh Edition of the TNM Classification for Lung Cancer. J. Thorac. Oncol..

[7] Asamura H., Nishimura K.K., Giroux D.J., Chansky K., Hoering A., Rusch V., Rami-Porta R., Araujo L.H., Beer D., Bertoglio P. (2023). IASLC Lung Cancer Staging Project: The New Database to Inform Revisions in the Ninth Edition of the TNM Classification of Lung Cancer. J. Thorac. Oncol..

[8] Nicholson A.G., Chansky K., Crowley J., Beyruti R., Kubota K., Turrisi A., van Meerbeeck J., Rami-Porta R., Goldstraw P., Asamura H. (2016). The International Association for the Study of Lung Cancer Lung Cancer Staging Project: Proposals for the Revision of the Clinical and Pathologic Staging of Small Cell Lung Cancer in the Forthcoming Eighth Edition of the TNM Classification for Lung Cancer. J. Thorac. Oncol..

[9] Chiappetta M., Lococo F., Leuzzi G., Sperduti I., Petracca-Ciavarella L., Bria E., Mucilli F., Filosso P.L., Ratto G.B., Spaggiari L. (2020). External validation of the N descriptor in the proposed tumour-node-metastasis subclassification for lung cancer: The crucial role of histological type, number of resected nodes and adjuvant therapy. Eur. J. Cardiothorac. Surg..

[10] Gebhart F. Plenary 4 Offers Early Look at 9th Edition of TNM Staging Classification for Thoracic Cancers. https://www.ilcn.org/plenary-4-offers-early-look-at-9th-edition-of-tnm-staging-classification-for-thoracic-cancers/.

[11] Wang R.-R., Li M.-J., Peng Q., Huang Z.-Y., Wu L.-L., Xie D. (2024). Validation of the 9th edition of the TNM staging system for non-small cell lung cancer with lobectomy in stage IA-IIIA. Eur. J. Cardiothorac. Surg..

[12] Nakao M., Suzuki A., Ichinose J., Matsuura Y., Okumura S., Ninomiya H., Mun M. (2024). Prognostic impact of the N2 subclassification and stage migration in the ninth edition of the TNM classification in surgically resected lung cancer. Lung Cancer.

[13] Kim S., Ahn Y., Lee G.D., Choi S., Kim H.R., Kim Y., Kim D.K., Park S., Yun J.K. (2025). Validation of the 9th Tumor, Node, and Metastasis Staging System for Patients with Surgically Resected Non-Small Cell Lung Cancer. Eur. J. Cancer.

[14] Mountain C.F. (1997). Revisions in the International System for Staging Lung Cancer. Chest.

[15] Huang J., Osarogiagbon R.U., Giroux D.J., Nishimura K.K., Bille A., Cardillo G., Detterbeck F., Kernstine K., Kim H.K., Lievens Y. (2023). The International Association for the Study of Lung Cancer Staging Project for Lung Cancer: Proposals for the Revision of the N Descriptors in the Forthcoming Ninth Edition of the TNM Classification for Lung Cancer. J. Thorac. Oncol..

[16] Uprety D., Mandrekar S.J., Wigle D., Roden A.C., Adjei A.A. (2020). Neoadjuvant Immunotherapy for NSCLC: Current Concepts and Future Approaches. J. Thorac. Oncol..

[17] Shalata W., Jacob B.M., Agbarya A. (2021). Adjuvant Treatment with Tyrosine Kinase Inhibitors in Epidermal Growth Factor Receptor Mutated Non-Small-Cell Lung Carcinoma Patients, Past, Present and Future. Cancers.

[18] Son J.W., Lee J., Jeon J.H., Cho S., Jung W., Shih B.C.-H., Kim K., Jheon S. (2024). Validation of IASLC 9th edition TNM classification for lung cancer: Focus on N descriptor. BMC Cancer.

[19] Chen K., Chen H., Yang F., Sui X., Li X., Wang J. (2017). Validation of the Eighth Edition of the TNM Staging System for Lung Cancer in 2043 Surgically Treated Patients With Non–small-cell Lung Cancer. Clin. Lung Cancer.

[20] Hishida T., Miyaoka E., Yokoi K., Tsuboi M., Asamura H., Kiura K., Takahashi K., Dosaka-Akita H., Kobayashi H., Date H. (2016). Lobe-Specific Nodal Dissection for Clinical Stage I and II NSCLC: Japanese Multi-Institutional Retrospective Study Using a Propensity Score Analysis. J. Thorac. Oncol..

[21] Detterbeck F.C., Woodard G.A., Bader A.S., Dacic S., Grant M.J., Park H.S., Tanoue L.T. (2024). The Proposed Ninth Edition TNM Classification of Lung Cancer. Chest.

[22] National Comprehensive Cancer Network (2024). NCCN Clinical Practice Guidelines in Oncology Version 1. https://www.nccn.org/guidelines/recently-published-guidelines.

[23] Yoshino I., Yoshida S., Miyaoka E., Asamura H., Nomori H., Fujii Y., Nakanishi Y., Eguchi K., Mori M., Sawabata N. (2012). Surgical Outcome of Stage IIIA- cN2/pN2 Non–Small-Cell Lung Cancer Patients in Japanese Lung Cancer Registry Study in 2004. J. Thorac. Oncol..

[24] Scherpereel A., Martin E., Brouchet L., Corre R., Duruisseaux M., Falcoz P.E., Giraud P., Pechoux C.L., Wislez M., Alifano M. (2023). Reaching multidisciplinary consensus on the management of non-bulky/non-infiltrative stage IIIA N2 non-small cell lung cancer. Lung Cancer.

[25] Forde P.M., Spicer J., Lu S., Provencio M., Mitsudomi T., Awad M.M., Felip E., Broderick S.R., Brahmer J.R., Swanson S.J. (2022). Neoadjuvant nivolumab plus chemotherapy in resectable lung cancer. N. Engl. J. Med..

[26] Wakelee H., Liberman M., Kato T., Tsuboi M., Lee S.-H., Gao S., Chen K.-N., Dooms C., Majem M., Eigendorff E. (2023). Perioperative Pembrolizumab for Early-Stage Non–Small-Cell Lung Cancer. N. Engl. J. Med..

[27] Heymach J.V., Harpole D., Mitsudomi T., Taube J.M., Galffy G., Hochmair M., Winder T., Zukov R., Garbaos G., Gao S. (2023). Perioperative durvalumab for resectable non-small-cell lung cancer. N. Engl. J. Med..

[28] Kim I.H., Lee G.D., Choi S., Kim H.R., Kim Y.-H., Kim D.K., Park S.-I., Yun J.K. (2024). Validation Study for the N Descriptor of the Newly Proposed Ninth Edition of the TNM Staging System Proposed by the International Association for the Study of Lung Cancer. J. Thorac. Oncol..

[29] Li S., Yan S., Lu F., Lv C., Wang Y., Li X., Wang Y., Yang Y., Wu N. (2021). Validation of the 8th Edition Nodal Staging and Proposal of New Nodal Categories for Future Editions of the TNM Classification of Non-Small Cell Lung Cancer. Ann. Surg. Oncol..

