# Supplementary material for: Validation of the 9th Edition of the TNM Classification in Patients with NSCLC and Lymph Node Involvement: A Retrospective, Multicentric, Observational Study
Source: Cancers (Basel). 2026 Feb 20;18(4):702. doi: 10.3390/cancers18040702 (PMC12939885; doi:10.3390/cancers18040702)
Supplement: Supplementary file 1 [file cancers-18-00702-s001.zip › cancers-4130408-supplementary.pdf]

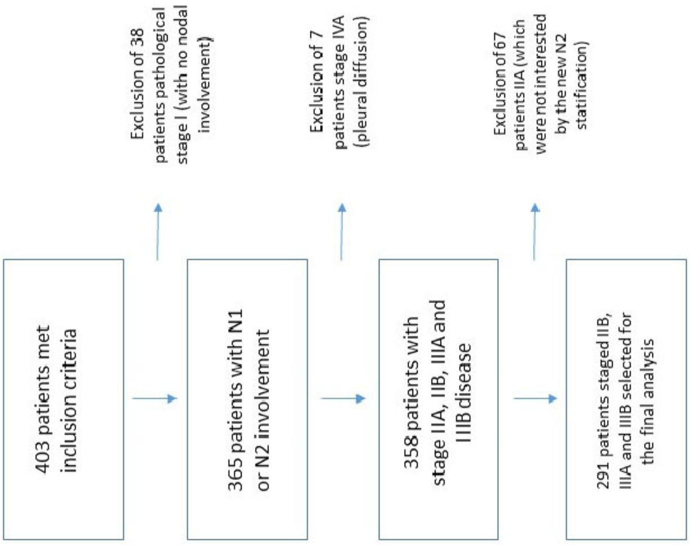

Supplementary figure S1. CONSORT diagram for patient selection

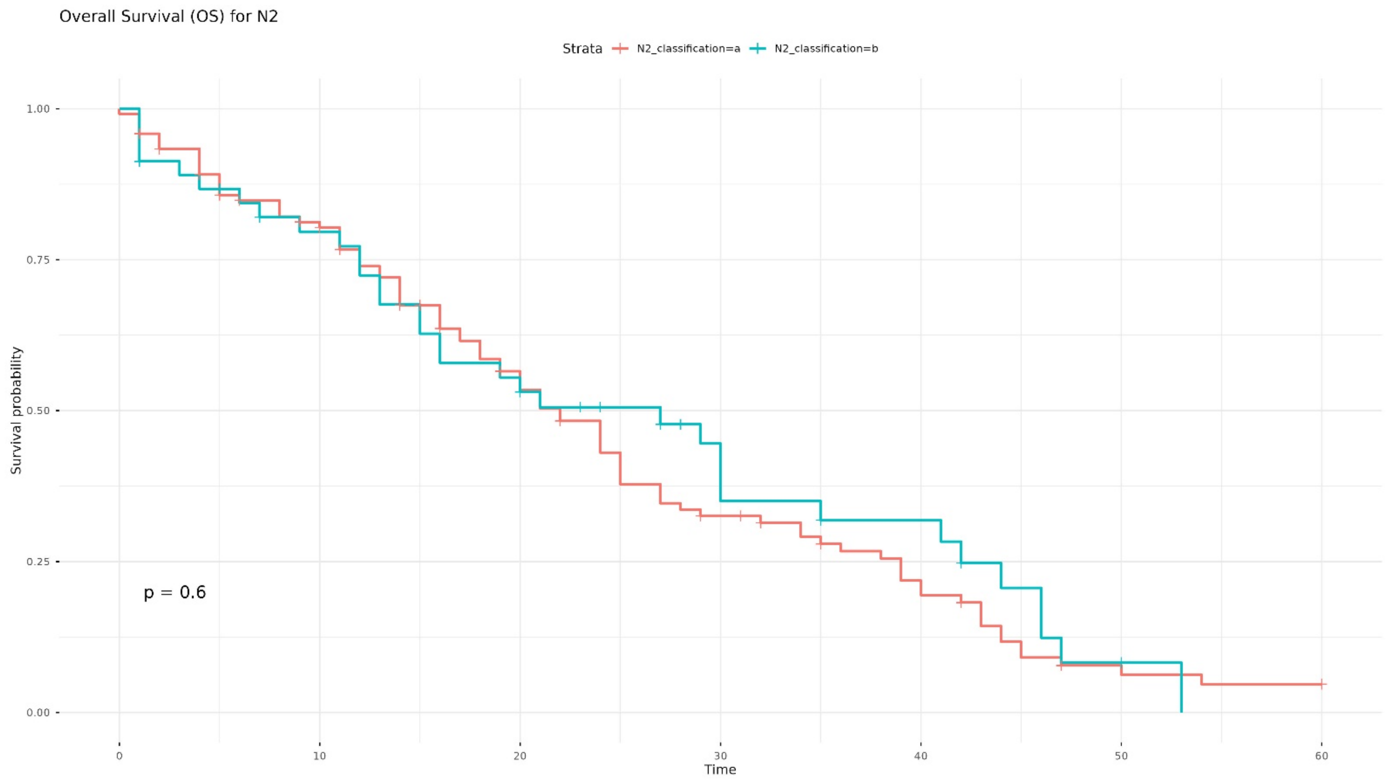

Supplementary figure S2. K-M curve for OS N2a vs N2b

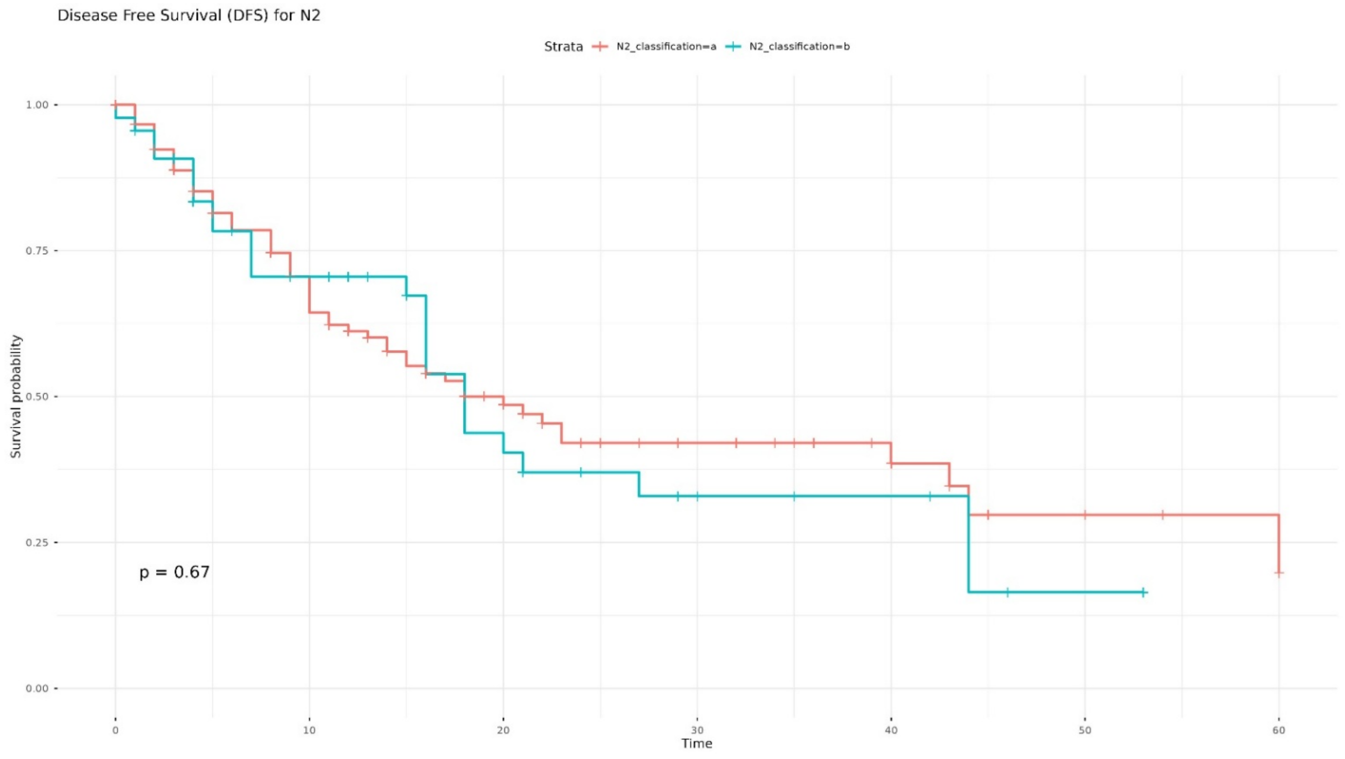

Supplementary figure S3. K-M curve for DFS N2a vs N2b

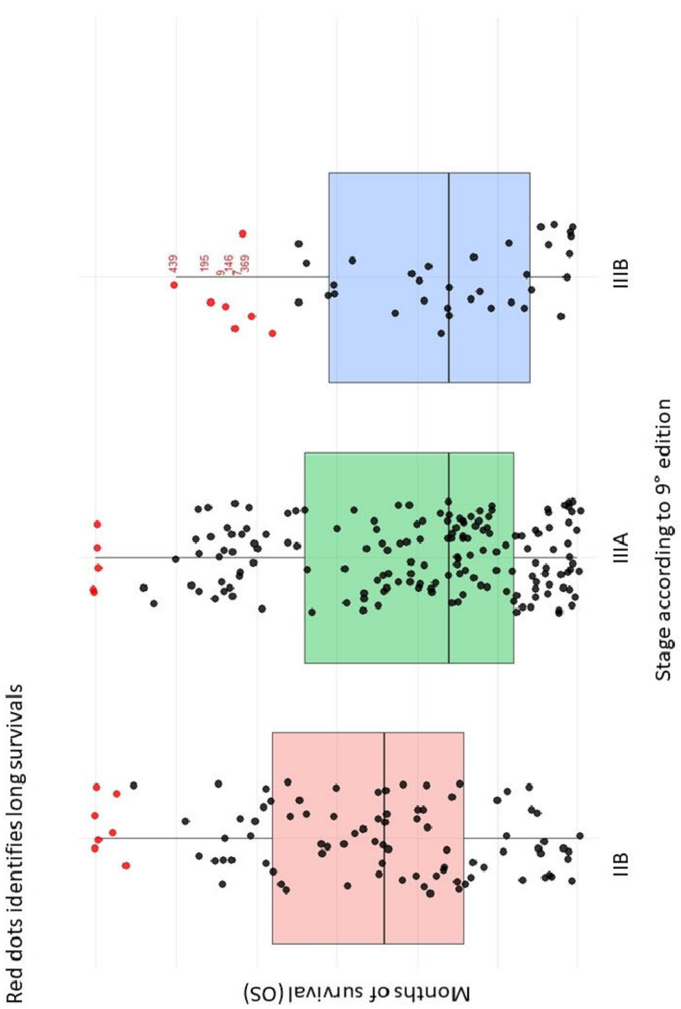

Supplementary Figure S4. boxplot per long survival dispersion
